# Sessile dislocations by reactions in NiAl severely deformed at room temperature

**DOI:** 10.1016/j.jallcom.2014.09.226

**Published:** 2015-02-05

**Authors:** D. Geist, C. Gammer, C. Rentenberger, H.P. Karnthaler

**Affiliations:** a University of Vienna, Faculty of Physics, Physics of Nanostructured Materials, 1090 Vienna, Austria; b National Center for Electron Microscopy, Lawrence Berkeley National Laboratory, Berkeley, CA 94720, USA

**Keywords:** High pressure torsion, B2 intermetallic compound, Transmission electron microscopy, 3D dislocation networks, Deformation-induced embrittlement

## Abstract

B2 ordered NiAl is known for its poor room temperature (RT) ductility; failure occurs in a brittle like manner even in ductile single crystals deforming by single slip. In the present study NiAl was severely deformed at RT using the method of high pressure torsion (HPT) enabling the hitherto impossible investigation of multiple slip deformation. Methods of transmission electron microscopy were used to analyze the dislocations formed by the plastic deformation showing that as expected dislocations with Burgers vector a〈100〉 carry the plasticity during HPT deformation at RT. In addition, we observe that they often form a〈110〉 dislocations by dislocation reactions; the a〈110〉 dislocations are considered to be sessile based on calculations found in the literature. It is therefore concluded that the frequently encountered 3D dislocation networks containing sessile a〈110〉 dislocations are pinned and lead to deformation-induced embrittlement. In spite of the severe deformation, the chemical order remains unchanged.

## Highlights

• HPT of NiAl enables the hitherto impossible study of multiple slip deformation at RT.
• TEM shows that glissile *a*〈1 0 0〉 dislocations react and form sessile *a*〈1 1 0〉 ones.
• 3D dislocation networks with sessile *a*〈1 1 0〉 cause deformation-induced embrittlement.
• By HPT chemical order is hardly reduced even in regions made nanocrystalline.

## 1 Introduction

The B2 ordered intermetallic compound NiAl is a candidate for high-temperature structural applications; still, poor room temperature (RT) ductility severely limits its fields of use [1]. Increases in ductility of NiAl were reported for elevated temperatures [2], [3], [4], whereas for RT, significant increases in ductility are usually not observed [5].

The dislocation characteristics in NiAl discussed in the literature can be classified in two different groups: (1) *“Soft” orientations* (basically all orientations that exhibit a non-zero resolved shear stress for a〈100〉{hkl} slip). For tensile and compressive tests at RT there is a broad consensus that the Burgers vector $\overset{\to }{b}$ of the glide dislocations is a〈100〉; other $\overset{\to }{b}$ have not been encountered [3], [6], [7], [8], [9], [10], [11], [12]. At RT the ductility is virtually always very low (most reports in the literature indicate values from abrupt brittleness up to 2.5%) [3], [13], [14]. This holds even for single crystals [3], [14]; still, there is a remarkable exception that it was shown that large tensile elongations (∼25%) can be obtained in high purity prestrained electropolished tension samples oriented for single slip [15]. They deform by single slip in a stage I (of the work-hardening curve) like way and even after these high elongations the failure occurs in a brittle like manner; therefore, the authors conclude that the brittle behavior of NiAl is caused by its inability to deform in the presence of stress concentrations [15], [16]. Also at temperatures above RT, a〈100〉 are prevailing in soft orientations [3]. (2) *“Hard” orientations* (deformation axis along 〈100〉). In this case, a〈111〉{hkl} slip was observed in compressive tests at low temperatures (77–600 K) [3]. For tensile tests at RT, immediate fracture after the elastic regime was reported [14]. At higher temperatures (above 600 K), glide of a〈111〉 and a〈110〉 dislocations was observed (e.g. [11], [9]). For these experiments, the deformation axis has to be set up very accurately (within ∼3° misorientation from 〈100〉); at larger misorientations, slip of a〈111〉 and a〈110〉 dislocations is replaced by a〈100〉{hkl} slip [11].

Therefore, it seems important to get a deeper insight into the microstructural evolution of NiAl in order to understand the difficulties in the plastic deformation of NiAl at RT. In the present study, the method of high pressure torsion (HPT) [17] is used to deform NiAl at RT since the high hydrostatic pressure of HPT as a constraint should have the advantage of suppressing the catastrophic brittleness. For example, even brittle B2 intermetallics (e.g. FeAl) were deformed by HPT leading to a reduction of their grain size and their long-range order [18]. Furthermore the HPT deformation of NiAl should make it possible to study multiple slip, thereby overcoming the hitherto experienced limitations of deformed NiAl specimens showing single slip only. To facilitate the analysis of the dislocations caused by HPT deformation of NiAl at RT, systematic transmission electron microscopy (TEM) studies are carried out in this paper based on images taken with different diffraction vectors $\overset{\to }{g}$ to analyze the dislocation reactions [19]. The results of the dislocation analysis are compared with elaborate calculations found in the literature that, using an embedded atom model, predict the core properties, the motion and the energies of dislocations in NiAl [20], [21]. In addition, the structural refinement resulting from HPT deformation at RT and its influence on the B2 long-range order are studied and compared with the findings in other intermetallic compounds deformed by HPT [22], [23].

## 2 Experimental procedure

From B2 ordered NiAl single crystals (Ni–49.5 at%Al), disk-shaped samples (6 mm in diameter and ∼0.5 mm thick) were prepared by wire sawing and subsequent grinding. They were deformed by HPT for up to 22 turns at RT applying a pressure of 8 GPa. After 22 turns, the deformation corresponds to a nominal shear strain γ≈830 for the rim of the disk. To reduce heating of the samples, a low deformation speed of 0.2 rotations per minute was chosen. For all samples deformed, an acoustic emission was noticed at the end of the deformation, in the moment of pressure release. Since the samples showed some macroscopic cracks after deformation, it is likely that the cracks were formed in the moment of pressure release. TEM samples with foil normals (FN) parallel to the torsion axis were prepared by mechanical polishing, dimpling and subsequent ion milling. TEM investigations were conducted at 200 kV. TEM bright field images, diffraction patterns and especially dark field (DF) images were taken to analyze the dislocations. The DF images were set up as two-beam cases far off the Bragg condition to reduce the width of the dislocation contrast [24]. Diffraction patterns were converted into radial intensity profiles using the PASAD software [18] to locally compare the size of coherently scattering domains (CSD) and ordered domains using the Williamson–Hall plot [25].

## 3 Results

### 3.1 3D dislocation networks formed by dislocation reactions

**Fig. 1:**
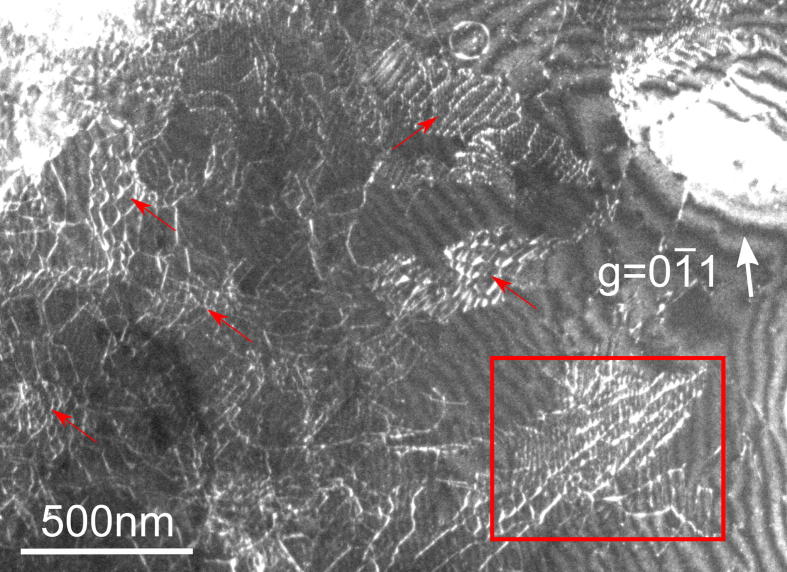
TEM DF image of a NiAl sample deformed by HPT for 6 turns. A dense arrangement of 3D dislocation networks (some are marked by red arrows) can be seen. The boxed area indicates the dislocation network analyzed in Fig. 3. (For interpretation of the references to color in this figure legend, the reader is referred to the web version of this article.)

Fig. 1 shows a region in an HPT sample deformed for 6 turns. Numerous 3D dislocation networks (some are marked by red arrows) are visible. The networks are formed by three different families of dislocations with different $\overset{\to }{b}$ that are entangled with each other (it should be noted that in some networks, not all families of dislocations are visible since dislocations only show contrast in the image if one of the visibility criteria is fulfilled: $\overset{\to }{g}\cdot \overset{\to }{b}\;\neq \;0$ or $\overset{\to }{g}\cdot (\overset{\to }{b}\times \overset{\to }{u})\;\neq \;0$ where $\overset{\to }{u}$ is the line direction of the dislocation [26]).

**Fig. 2:**
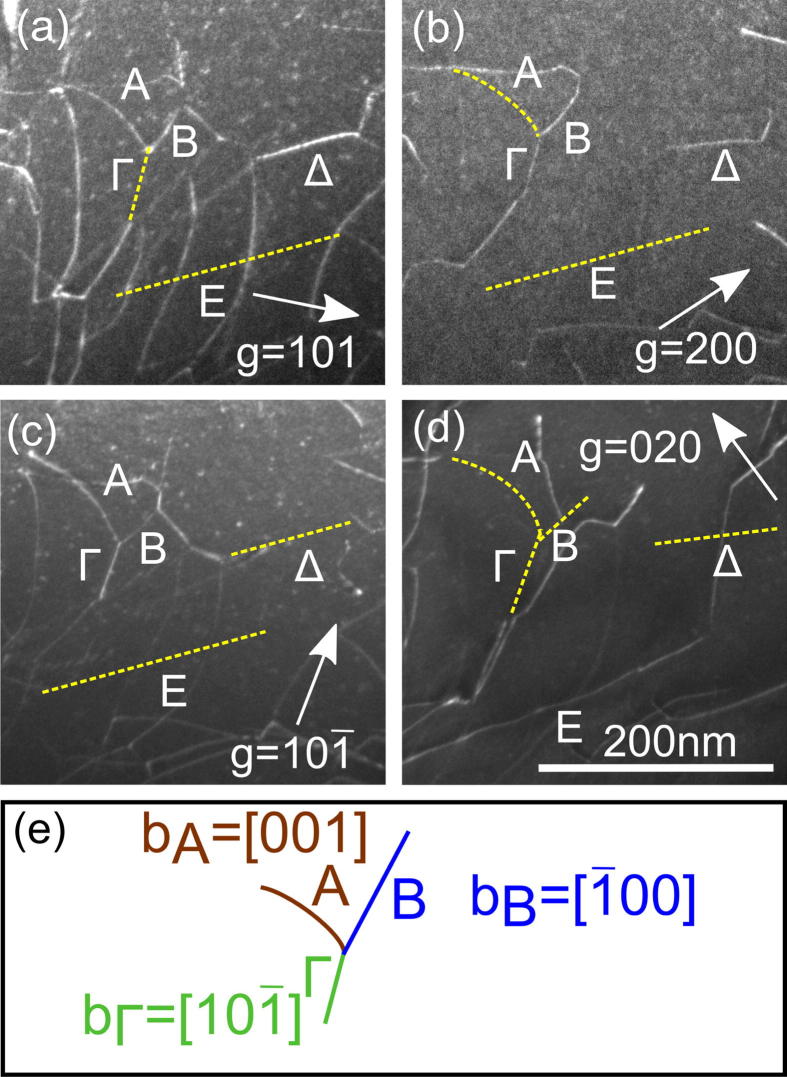
Dislocation analysis in NiAl deformed by HPT (6 turns). (a–d) TEM DF images (FN∼[012]). (a–c) BD∼[010] and (d) BD∼[001]. The result of the analysis is shown by the letters labeling the dislocations (Latin for $\overset{\to }{b}=a\langle 1\;0\;0\rangle$ and Greek for $\overset{\to }{b}=a\langle 1\;1\;0\rangle$). If a certain dislocation is out of contrast in an image, its approximate position is indicated by a dashed yellow line. (e) Sketch of three dislocations (A, B, Γ) forming a node ($\overset{\to }{b}$ indicated). (For interpretation of the references to color in this figure legend, the reader is referred to the web version of this article.)

In Fig. 2, a region is imaged with dislocations that are not entangled in a dense network (the sample was HPT deformed for 6 turns); $\overset{\to }{g}$ and the beam directions (BD) are indicated. Based on the visibility criteria and the restriction that the dislocations must be either of a〈100〉,a〈110〉 or a〈111〉 type, it is concluded from the different contrast of the dislocations in Fig. 2(a)–(d) that the Burgers vectors of the dislocations marked with Latin letters (A, B and E) are of a〈100〉 type whereas the dislocations marked with Greek letters (Γ and Δ) are of a〈110〉 type. The Burgers vectors of the dislocations forming a threefold node are shown in the sketch of Fig. 2(e). Dislocations A, B and Γ are reacting in a common node, which is consistent with $\sum {\overset{\to }{b}}_{i}=0$.

**Fig. 3:**
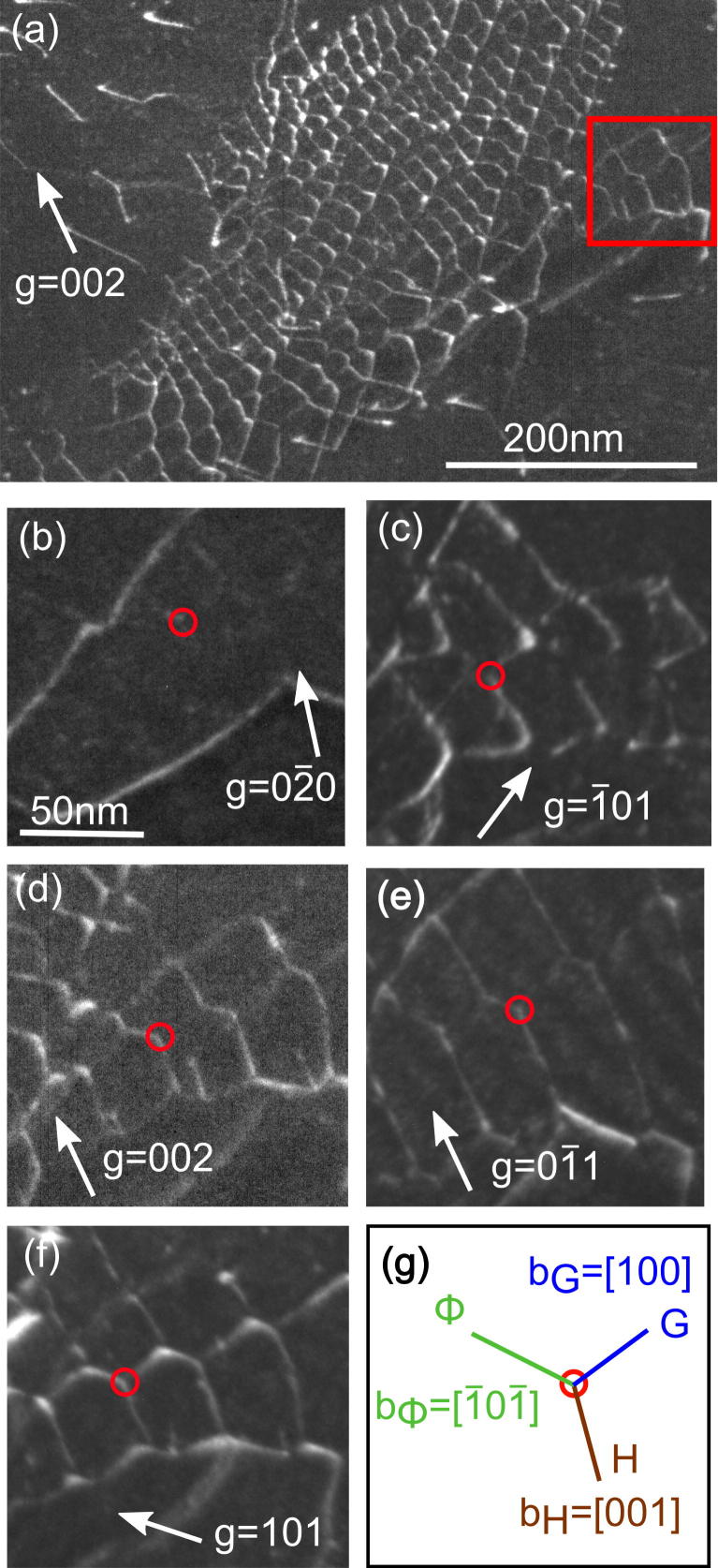
Analysis of the $\overset{\to }{b}$ of the dislocations forming a dense 3D network (cf. the marked region in Fig. 1); TEM DF images, FN ∼ [0 1 2]. (a) Overview of the network, the red rectangle indicates the area that is analyzed in (b–f). In (a–f), the $\overset{\to }{g}$ are indicated and BD ∼ [0 1 0], [0 0 1], [0 1 0], [0 1 0], [0 1 1], [0 1 0] respectively. The red circles denote corresponding positions in the network. (g) Sketch of a dislocation node of the network with the resulting $\overset{\to }{b}$. (For interpretation of the references to color in this figure legend, the reader is referred to the web version of this article.)

Fig. 3 demonstrates the analysis of a dense 3D dislocation network (marked by the red rectangle in Fig. 1). In Fig. 3(a) the whole network is imaged. Fig. 3(b)–(f) shows a region of the network under different diffraction conditions to determine the Burgers vectors of the dislocations. The network is built up by threefold nodes as repeating element. In Fig. 3(g), such a node is sketched. It can clearly be seen that the contrast for dislocation Φ (indicated in Fig. 3(g)) vanishes for $\overset{\to }{g}=[0\;\overset{¯}{2}\;0]$ and $\overset{\to }{g}=[\overset{¯}{1}\;0\;1]$, therefore from $\overset{\to }{b}\|[0\;\overset{¯}{2}\;0]\times [\overset{¯}{1}\;0\;1]$ it is concluded: ${\overset{\to }{b}}_{\Phi }=a[\overset{¯}{1}\;0\;\overset{¯}{1}]$. For dislocation G, the contrast vanishes for $\overset{\to }{g}=[0\;0\;2]$ and $\overset{\to }{g}=[0\;\overset{¯}{1}\;1]$ leading to the result: ${\overset{\to }{b}}_{G}=a[1\;0\;0]$. For dislocation H it can be concluded from Fig. 3(b)–(d) and (f) that ${\overset{\to }{b}}_{H}=a[0\;0\;1]$. Like in Fig. 2, the equation for dislocations reacting in a common node $\sum {\overset{\to }{b}}_{i}=0$ is fulfilled for the dense network.

**Fig. 4:**
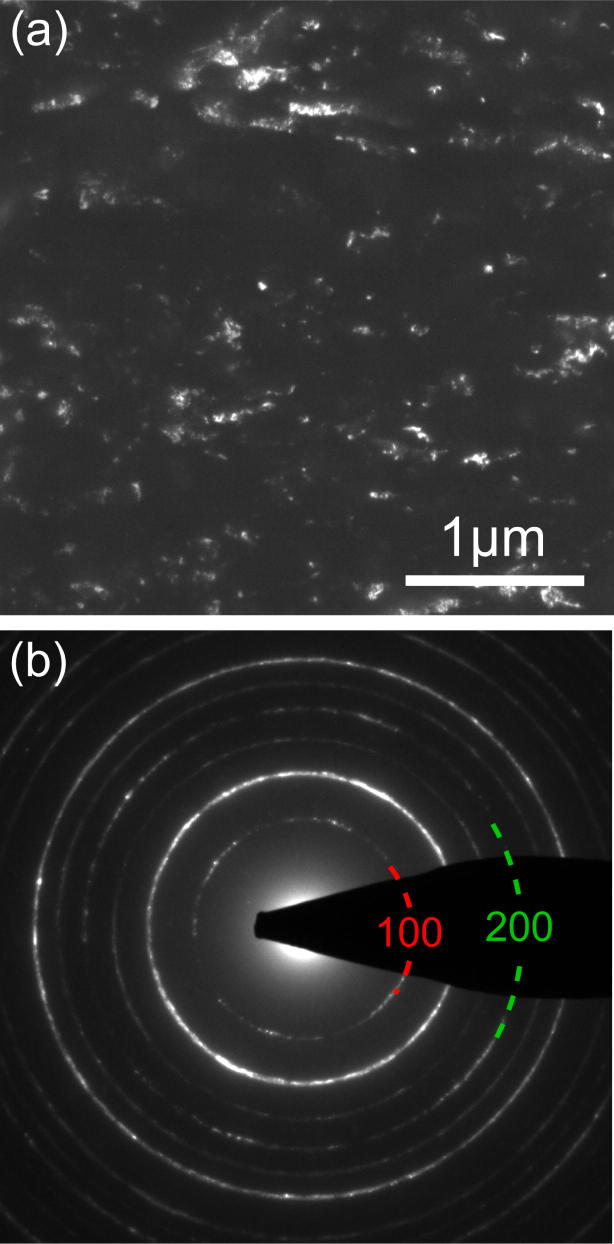
NiAl deformed by HPT (22 turns). (a) TEM dark field image revealing an ultra-fine grained/nanocrystalline structure. (b) Selected area diffraction pattern of the region shown in (a).

Fig. 4 shows a DF image (near Bragg condition) and a diffraction pattern of NiAl deformed for 22 turns. Fig. 4(a) reveals an ultra-fine grained/nanocrystalline structure with grain sizes varying from 10 nm to 1 μm. In the selected area diffraction pattern (cf. Fig. 4(b)) taken from the area shown in (a), in addition to the fundamental (2 0 0) reflection ring, (1 0 0) superlattice reflections are clearly visible (indicated by green and red segments of the corresponding rings, respectively). This means that the material is still chemically ordered.

**Fig. 5:**
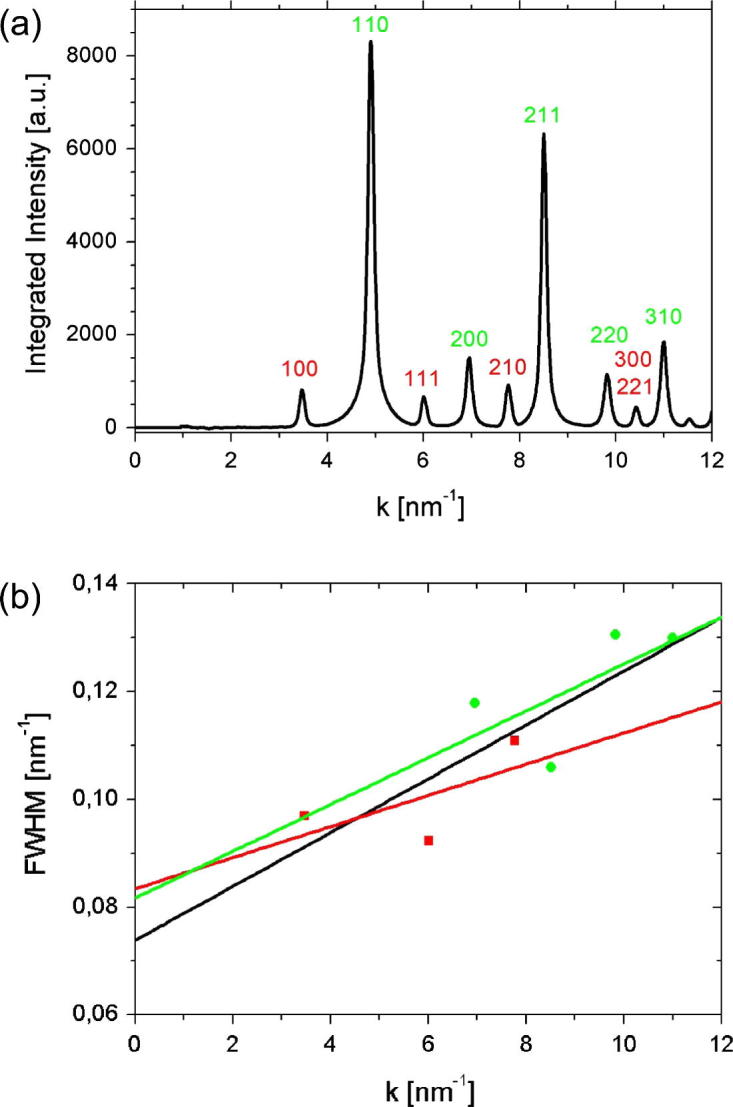
Analysis of the DP of NiAl deformed by 22 turns by HPT (Fig. 4(b)). Fundamental and superlattice reflections are represented in green and red, respectively. (a) Radial intensity distribution obtained by azimuthal integration of the diffraction pattern (Fig. 4(b)) with the PASAD software (background-subtracted). After the severe deformation, the chemical order is still present as the intensity of the (1 0 0) ring is very well detectable. (b) Williamson–Hall plot of the radial intensity distribution shown in (a) for fundamental (green), superlattice (red) and all (black) reflections. (For interpretation of the references to color in this figure legend, the reader is referred to the web version of this article.)

Fig. 5(a) shows the intensity profile obtained by azimuthal integration of Fig. 4(b) (fundamental and superlattice reflections are marked in green and red, respectively). In Fig. 5(b), a Williamson–Hall plot [25] of the data is shown, that yields a volume weighted mean CSD size of about 12nm (black fit). For this analysis, the (1 1 0), (2 2 1) and (3 0 0) reflections were not considered because the FWHM of the (1 1 0) seems to be biased by saturation effects of the CCD camera and the (2 2 1) and (3 0 0) cannot be separated due to their identical lattice spacing and therefore identical *k*-value. For the present study, it is important that an analysis of fundamental reflections and superlattice reflections in separate Williamson–Hall plots yields CSD sizes that are within the error margins of their combined analysis. This means that the CSD size and the size of the ordered domains do not differ significantly, indicating a chemically highly ordered phase.

## 4 Discussion

### 4.1 Dislocation characteristics and the observed dislocation reaction

In NiAl, the occurrence of dislocations other than a〈100〉 was in both tensile and compression tests at RT only observed in the hard orientation (deformation axis ∼〈100〉 and therefore zero resolved shear stress for a〈100〉{hkl} slip). For shear deformation experiments (including HPT), the resolved shear stress for a〈100〉{hkl} slip becomes zero for shear directions 〈110〉 with the shear plane normal being the 〈110〉 direction normal to the shear direction (e.g. shear direction [110] and shear plane $\left(1\;\overset{¯}{1}\;0\right)$)[27]. Since both the areas investigated in Fig. 1, Fig. 2, Fig. 3 are more than 10° inclined to that situation and due to the very low Peierls stress at RT of the a〈100〉 dislocation as compared to all other possible types of dislocations [20], all the regions investigated can be considered as being in soft orientation. This means that our observations of a〈100〉 dislocations are in accordance with literature.

In addition to the a〈100〉 dislocations that are expected, the TEM analysis (cf. Fig. 2, Fig. 3) shows the occurrence of a〈110〉 dislocations that are not expected at RT deformation. Since the latter are always connected to threefold nodes, we conclude that they are formed by the reaction of two a〈100〉 dislocations. In this context, it should be mentioned that based on simple reasoning, two dislocations (${\overset{\to }{b}}_{1}$ and ${\overset{\to }{b}}_{2}$) with orthogonal $\overset{\to }{b}$ should neither attract nor repel each other. Also, when Frank’s dissociation rule is applied that the energy of a dislocation is proportional to ${\overset{\to }{b}}^{2}$, the energy $E_{R}$ of a dislocation formed by the reaction of two dislocations with orthogonal $\overset{\to }{b}$ would be just the sum of the energies of the reacting dislocations: $E_{R}\propto {\overset{\to }{b}}_{R}^{2}={\overset{\to }{b}}_{1}^{2}+{\overset{\to }{b}}_{2}^{2}$ [28]. When isotropic and especially anisotropic elasticity theory are taken into account the situation is different. In the present case of the intermetallic compound NiAl the value of the Zener anisotropy ratio $A=(2C_{44})/(C_{11}-C_{12})$ is quite high (A≈3.7 using the experimentally determined elastic constants from [29], A≈3.2 according to the calculations in [30]). This high value plus the influence of the different core structures arising in a〈100〉 and a〈110〉 dislocations were considered in elaborate calculations for NiAl. The published results show that the reaction of a〈100〉 dislocations with parallel line directions forming a〈110〉 dislocations can lead to a considerable reduction of the line energy (=elastic energy + core energy) of 1eV/Å to 2eV/Å [20], [21] where the line energy of an a〈100〉 dislocation is about 2.6–3.7 eV/Å, depending on the character of the dislocation.

The relatively high density of a〈110〉 dislocations found in the present study and the fact that all of them are entangled in threefold nodes demonstrates that the dislocation arrangements formed during HPT facilitate the reaction of a〈100〉 dislocations leading to a〈110〉 dislocations. This reaction has not been observed so far in NiAl deformed at RT. We propose that by severe plastic deformation by HPT for 6 turns (γ≈230), glide dislocations of different a〈100〉 type $\overset{\to }{b}$ that are gliding on different glide planes are activated which can react and form a〈110〉 dislocations.

The reactions leading to a〈110〉 dislocations that were found in previous studies were observed at high temperatures only where a〈110〉 dislocations are mobile and contribute to the plastic deformation. In material deformed at elevated temperatures (800–900 K), a〈100〉 are frequently observed. In addition, a〈110〉 are also encountered and are believed to be reaction products [31]. Results that were achieved in the temperature range 850–990 K indicate that a〈110〉 are dominating [32].

### 4.2 Comparison of the mobility of glide dislocations with that of sessile reaction dislocations

The fact that no free a〈110〉 dislocations are observed but all of them are reaction products of intersecting a〈100〉 dislocations (cf. Fig. 2, Fig. 3) indicates that a〈110〉 dislocations have a very low mobility at RT.

Our observations are therefore in agreement with the sophisticated calculation of the core properties and predictions of the mobility of dislocations in NiAl as found in the literature [20], [21]. These calculations, which do not include thermally activated processes, show that a〈100〉 dislocations are expected to glide on the close packed {110} planes since they have glide dissociated cores on the {110} planes giving rise to a moderate Peierls stress. In contrast to the glissile a〈100〉 dislocations, the dislocations with $\overset{\to }{b}=a\langle 1\;1\;0\rangle$ are considered to be sessile up to high stresses as was deduced from molecular static calculations [20] and from atomic resolution studies combined with simulated images based on anisotropic elasticity calculations of the dislocation cores [9].

The a〈110〉 dislocations show a climb dissociation (of about 0.4 nm) into two 〈100〉 edge dislocations which reduces their total energy by ∼0.15eV/Å as compared to that of the complete a〈110〉{110} dislocation [20]. It is proposed that this dissociation causes a high Peierls stress for the a〈110〉 dislocations that is about two orders of magnitude higher than that for the a〈100〉 dislocations. Therefore, it is concluded from the calculations [20] that the a〈110〉 dislocations are sessile and do not contribute to the plastic deformation at RT which is in agreement with the present results.

### 4.3 Comparison of the present reaction dislocations with Lomer Cottrell dislocations in fcc explaining the different situation

The formation of sessile dislocations by the reaction of glissile ones as encountered in this study of HPT deformed NiAl can be compared with dislocation reactions in fcc metals and alloys. There, the initial situation seems to be similar: The mobile dislocations $\frac{a}{2}\langle 1\;1\;0\rangle \{1\;1\;1\}$ are glide dissociated into Shockley partials bounding a stacking fault on {111}. When glide dislocations moving on two different {111} planes react, a so called Lomer Cottrell (LC) dislocation is formed which lies along the intersection line of the two {111} glide planes. The LC dislocations have a non-planar dissociation as they consist of three partials and two stacking faults on intersecting {111} planes. In this configuration the LC dislocations cannot move and are considered to be sessile. In contrast, it was found that the LC dislocations can be constricted by moderate stresses at RT, e.g. a stress arising at the end of stage II of the work-hardening curve of single crystals oriented for single slip [33]. It should be pointed out that this transition from the “locked” LC configuration to that of a glissile Lomer dislocation was found to occur at RT not only in fcc metals [33] but also in Cu–15 at%Al, an fcc alloy of very low stacking fault energy [34]. Therefore, this common transition from the sessile LC configuration to that of a mobile Lomer dislocation bowing out on {100} planes has been put forward as an alternative explanation for stage III of the work-hardening curve since it can explain the reduced work hardening of stage III as compared to that of stage II [33]. This means the present situation in NiAl is completely different since the reaction product are a〈110〉 dislocations which are sessile with any dislocation character. They can not be mobilized at RT as the calculated stress (2–6 GPa) that is needed to make them mobile exceeds the fracture stress [13], [35].

### 4.4 Correlation between sessile reaction dislocations and cracking

The 3D dislocation networks that are frequently formed during HPT deformation (cf. Fig. 1) contain in every threefold node a〈110〉 dislocations that are not glissile (cf. Fig. 3). The networks do not extend to the grain boundaries but end within the grain (cf. Fig. 1). Taking into account the Frank criterion for the presence of long-range stresses [27], it becomes clear that internal stresses have to be present: two different routes $R_{1}$ and $R_{2}$ which are completely within one grain can be chosen to get from point *A* to point *B* in such a way that $R_{1}$ involves crossing a dislocation network while $R_{2}$ does not. Since $R_{1}$ and $R_{2}$ have the same starting point and end point, the misorientation caused by the dislocation network on $R_{1}$ has to be compensated by internal stresses either on $R_{1}$ or $R_{2}$. Therefore it is concluded that internal stresses are built up by the sessile networks and lead to deformation-induced embrittlement. It is proposed that this is the reason for the catastrophic failure of NiAl during deformation by usually only small amounts of strain (0–5%) at RT [3], [13], [14], [36], [37]. The big scatter of tensile and compressive ductility of single crystalline NiAl found in the literature can thus be explained by the deviation from single slip orientations since for single slip, no networks are expected to be formed at the beginning of the deformation (stage I of the work hardening curve of single crystals). This agrees well with the results found in the literature showing a TEM study of the evolution of substructures in deformed NiAl single crystals oriented for single slip [12]. The importance of single slip for the ductility also agrees with the fact that only stage I behavior has been encountered [15] since multiple slip leads as shown here to sessile dislocations causing stress concentrations. In addition, the roughness of the surface was proposed as an important parameter that can limit the tensile ductility of single crystalline NiAl samples [38]. By electropolishing the surfaces and using a single slip orientation with one Burgers vector being active, an increased tensile ductility was achieved [36], [38], [15]. This agrees well with our suggestion that deformation-induced embrittlement is caused by the activity of different Burgers vectors that are necessary to form sessile dislocations by reactions.

In contrast to tensile experiments the high quasi-hydrostatic pressure of 8 GPa applied during HPT deformation can give rise to a different scenario. A possibility is the formation of microcracks that do not transform into catastrophic cracks because the high pressure can lead to crack closure. But when the hydrostatic pressure is taken away, the elastic stresses built up in the sample during deformation will lead to the formation of cracks. This is confirmed by the experimental findings: An acoustic emission is heard during pressure release at the end of the HPT deformation and the samples show cracking that can be seen with the bare eye. In this context, it is proposed that the nano- and submicrometer voids and cracks observed in Cu and Cu–1 wt%Pb deformed by equal channel angular pressing [39], [40], [41] are caused by internal stresses built up during the severe plastic deformation which are released during unloading. As expected, the effect is much smaller in Cu than in an intermetallic compound since the dislocation tangles formed in fcc structures are much weaker obstacles and can release stresses with less cracking.

### 4.5 Preservation of long-range order even after severe plastic deformation by HPT

In a recent publication the hypothesis was put forward that chemical disordering by deformation is caused by glide dislocations containing distinct antiphase boundary (APB) faults [42]. This is supported by the findings in intermetallic compounds independent of their ordered structure, e.g. in Cu_3_Au, Ni_3_Al and in FeAl having L1_2_ and B2 long-range order, respectively [43], [22], [23]. In contrast, when the glide dislocations are dissociated with structural faults that do not change the chemical order, disordering by deformation is not expected to occur as it was observed in the case of Zr_3_Al where the glide dislocations contain superlattice intrinsic stacking faults [44]. Also, the present results of NiAl are in support of the above hypothesis. Even after severe plastic deformation by HPT (nominal shear strain γ≈830) leading to an ultrafine grained/nanocrystalline structure, the diffraction patterns reveal a high degree of order (cf. Fig. 4, Fig. 5). The fact that disordering by deformation is not observed is explained by the intrinsic properties of the dislocations that are active in the present case: The dislocations a〈100〉 (cf. Fig. 2) are perfect dislocations which means they are not dissociated and therefore do not contain APB faults. In this respect the present results can be compared with those of NiAl deformed by ball milling where disordering was not encountered either [45]. The lack of disordering observed in NiAl is in contrast to the results of deformation induced disordering reported for FeAl which is also a B2 ordered compound [46], [23]. In FeAl the active glide dislocations $\overset{\to }{b}=a\langle 1\;1\;1\rangle$ are dissociated according to the scheme $a\langle 1\;1\;1\rangle \to \frac{a}{2}\langle 1\;1\;1\rangle +\mathrm{APB}+\frac{a}{2}\langle 1\;1\;1\rangle$. The resulting storage of APB faults leads to a decrease of the chemically ordered domain size and finally to a pronounced reduction of long-range order [47]. The finding that the CSD sizes of fundamental reflections and those of the superlattice reflections are the same in NiAl (cf. Fig. 5) corroborates the result that only 〈100〉 dislocations are active up to high strains and no disordering is present.

The preservation of the B2 long-range order in NiAl is of specific importance for the mechanical behavior of NiAl deformed at RT. The absence of chemical disordering (that would lead to a transition from the B2 to the bcc structure) inhibits the activation of $\frac{a}{2}\langle 1\;1\;1\rangle h\;k\;l$ slip, which is the dominant slip system for virtually all bcc materials [48]. Therefore, the deformation-induced embrittlement as discussed above can only occur up to high amounts of strain if the chemical order is not affected by dislocation activity.

## 5 Conclusions

• HPT deformation of NiAl enables to carry out the up till now impossible investigation of multiple slip deformation at RT.
• The TEM study shows that the glissile a〈100〉 dislocations frequently form a〈110〉 dislocations by dislocation reactions. The a〈110〉 dislocations are considered to be sessile. This is in full agreement with the calculations predicting core properties, motion and energies of dislocations in NiAl [20], [21].
• 3D dislocation networks containing a〈110〉 dislocations are pinned and therefore lead to deformation-induced embrittlement. The occurrence of long-range internal stress concentrations is confirmed by the Frank criterion.
• During HPT deformation the high quasi-hydrostatic pressure of 8 GPa prevents the sample from catastrophic failure. It is therefore proposed that the cracks observed after deformation form in the moment of pressure release.
• By HPT deformation the chemical order is not significantly reduced even in nanocrystalline regions of the sample. This is explained by the lack of antiphase boundary dissociated dislocations. Therefore, the slip system does not change with increasing strain. This is crucial for the occurrence of the a〈100〉 glide dislocations and their reactions forming a〈110〉 sessile dislocations.
• Our results on sessile dislocation reactions are able to explain by an orientation dependence the scatter of the results of single crystal ductility of NiAl found in the literature.
